# Anti-IL-22 Antibody Attenuates Acute Graft-versus-Host Disease via Increasing Foxp3^+^ T Cell through Modulation of CD11b^+^ Cell Function

**DOI:** 10.1155/2018/1605341

**Published:** 2018-08-07

**Authors:** Jianbo Wu, Jian Gu, Shun Zhou, Hao Lu, Yunjie Lu, Ling Lu, Xuehao Wang

**Affiliations:** ^1^Department of Liver Surgery, First Affiliated Hospital of Nanjing Medical University, Key Laboratory of Living Donor Liver Transplantation, National Health and Family Planning Commission, Nanjing, Jiangsu Province, China; ^2^Department of General Surgery, Changzhou No. 2 People's Hospital Affiliated to Nanjing Medical University, Changzhou City, Jiangsu Province, China

## Abstract

Transfer of splenocytes isolated from B6 mice into normal B6D2F1 mice induces acute graft-versus-host disease (aGVHD), resulting in the expansion of donor cytotoxic T lymphocytes that eliminate recipient B cells. The cytokine IL-22, secreted by Th1 cells, Th17 cells, and innate immune cells, is structurally related to IL-10. To investigate the association between IL-22 and aGVHD, an anti-mouse IL-22 antibody (IL-22Ab) was used to ablate IL-22 activity in a mouse aGVHD model. Administration of IL-22Ab significantly reduced the progression of aGVHD in B6D2F1 recipients of B6 grafts. IL-22Ab treatment also decreased the percentage of interferon-*γ* ^+^ and tumor necrosis factor-*α* ^+^ T cells but increased the number of forkhead box p3^+^ regulatory T cells (Tregs). In the presence of Tregs and donor CD11b^+^ cells, IL-22Ab protected against aGVHD. *In vitro* Treg induction was more efficient when CD4^+^CD25^−^ T cells differentiated in the presence of CD11b^+^ cells obtained from IL-22Ab-treated GVHD mice, compared with cocultured untreated control cells. Finally, IL-22Ab modulated the expression of cytokines and costimulatory molecules in CD11b^+^ cells in aGVHD mice. We therefore conclude that IL-22Ab administration represents a viable approach for treating aGVHD.

## 1. Introduction

Interleukin- (IL-) 22, a member of the IL-10 family of cytokines, plays an important role in the pathogenesis of autoimmune diseases such as rheumatoid arthritis [1], psoriasis [2], and acute hepatitis [3] in humans. IL-22 also plays protective roles. During experimental colitis associated with inflammatory bowel disease [4], IL-22 functions in maintaining the integrity of the intestinal epithelium via signaling pathways that promote epithelial cell survival, proliferation, and wound healing. In addition, IL-22 induces the expression of proinflammatory cytokines that activate signal transducer and activator of transcription 3 (Stat3), which is associated with autoimmune diseases [5–7]. Several leukocyte subsets produce IL-22, including T-helper (Th) cells [8] and innate lymphoid cells [9]. However, expression of the IL-22 receptor (IL-22R) is restricted to nonhematopoietic stromal cells, including epithelial cells of the lung and gastrointestinal tract [10–12].

Graft-versus-host disease (GVHD) is a major complication of allogeneic hematopoietic stem cell transplantation [13], resulting in significant morbidity and mortality in organ transplant patients [14]. Current therapies for treating or controlling acute GVHD (aGVHD) have exhibited limited success [15]. The graft-versus-host reaction can be induced in inbred F1 mice by injecting spleen cells of parental origin [16] that generate donor CD8^+^ CTLs specific for host MHC I that eliminate host spleen cells, particularly B cells, within two weeks. This results in a lymphopenic state termed “acute GVHD in the absence of pathogen infection.”

Several recent studies showing that IL-22 deficiency attenuates murine aGVHD [17] and that IL-22 exhibits deleterious effects in an aGVHD model by promoting CD3^+^ T-cell infiltration [18] which demonstrated the importance of IL-22 in the pathogenesis of aGVHD. By contrast, another group reported that IL-22 protects intestinal stem cells during aGVHD [19]. In the present study, we examined the biological effects of an anti-IL-22 antibody (IL-22Ab) in a mouse model of aGVHD. Surprisingly, our results consistently showed that IL-22Ab strongly suppresses cytokine production, allogeneic cell expansion, and cytotoxic activity in treated mice. Mechanistic studies demonstrated that treatment with the IL-22Ab induces increased production of IL-10 and transforming growth factor- (TGF-) *β*, as well as costimulatory molecules of CD11b^+^ cells that induce expansion of Tregs both *in vivo* and *in vitro*. Our results strongly suggest that administration of the IL-22Ab could be effective for preventing and treating autoimmune diseases such as aGVHD [20].

## 2. Materials and Methods

### 2.1. Animals

C57BL/6 (H-2b) and B6D2F1 (H-2b/d) mice were purchased from Jackson Laboratory (Bar Harbor, ME). C57BL/6 Foxp3 knock-in mice were generously provided by Dr. Talil Chatilla (UCLA). We maintained breeding colonies in our animal facility. Mice were used at age of 8–12 weeks. All experiments using mice were performed in accordance with protocols approved by the Institutional Animal Care and Use Committees at Nanjing Medical University.

### 2.2. Cytokine Measurements

Blood samples and spleen homogenate were obtained from recipient mice at the indicated day point after cell transplantation. IL-22 levels were measured using commercially available enzyme-linked immunosorbent assay (ELISA) kits (BioLegend) according to the manufacturer's instructions.

IL-17, IFN-*γ*, IL-4, TNF-*α*, IL-10, IL-6, and TGF-*β* were measured using commercially available ELISA kits (R&D Systems, Minneapolis, MN).

### 2.3. Development of Mouse aGVHD Models

aGVHD was induced by the intravenous injection of 50 × 10^6^ splenocytes isolated from B6 mice into B6D2F1 mice as previously reported [21]. To maintain as much homogeneity of donor cell populations as possible, aGVHD was induced on the same day using cells processed simultaneously under the same conditions. After 2 weeks, mice were sacrificed, and the cells were measured by staining splenocytes with anti-mouse-H2kb and anti-mouse-H2kd antibody (recognizing donor cells) and cell lineage markers (BioLegend). In some experiments, CD11b^+^ cells were depleted using anti-PE CD11b and anti-PE beads from the B6 spleen cells.

### 2.4. Cell Isolation and Preparation

CD4^+^CD25^−^ T cells were isolated from spleen cells of aGVHD mice using a CD4^+^ T cell isolation kit (Miltenyi Biotec). CD11b^+^ cells were obtained from the spleens of anti-IgG- or IL-22Ab-treated aGVHD mice by positive selection, using anti-PE-CD11b and anti-PE beads through AutoMACS (Miltenyi Biotec). CD4^+^CD25^−^ T cells and CD11b^+^ cells were tested with >98% purity before cell culture.

In some experiments, CD4 cells were sorted using anti-PE CD4 and anti-PE beads from the spleen cells.

### 2.5. Quantitative Real-Time PCR

RNA was extracted from harvested cells using an RNA simple total RNA kit according to the manufacturer's instructions (Tiangen Biotech); cDNA was synthesized using RT-Master Mix (TaKaRa). The cDNA product was amplified by qRT-PCR in the ABI prism 7700 sequence-detection system (Applied Biosystem, Foster City, CA) with relevant primers for mice. Data were analyzed using the relative gene expression method and were normalized with GAPDH levels in the samples. The measurements of each sample were performed in triplicate. Primer sequences used for PCR amplification are as follows:

IL-22R1: forward primer—5-GCAAACAGAGAGAATACGAGTTCC-3; reverse primer—5-AGAAGGAGTAGGCCCACGTC-3

CD80: forward primer—5-TGCTGCTGATTCGTCTTTCAC-3; reverse primer—5-GAGGAGAGTTGTAACGGCAAG-3

CD86: forward primer—5-CTGGACTCTACGACTTCACAATG-3; reverse primer—5-AGTTGGCGATCACTGACAGTT-3

CD83: forward primer—5-CGCAGCTCTCCTATGCAGTG-3; reverse primer—5-GTGTTTTGGATCGTCAGGGAAT-3

MHC-II: forward primer—5-GATCGGATCCAACCCTGAGGATTCA-3; reverse primer—5-GATCGATCCTGTCCTCCCCTGGCAAGA-3

*β*-Defensin: forward primer—5-CTCTCGGGACACAATAAGCTCT-3; reverse primer—5-CAAGCCATAAAAGCAGGTTCTGA-3

Reg3*γ*: forward primer—5-ATGCTTCCCCGTATAACCATCA-3; reverse primer—5-ACTTCACCTTGCACCTGAGAA-3

### 2.6. Generation of CD4^+^ iTreg Cells Ex Vivo

CD4^+^CD25^−^ T cells were separated from normal F1 mice. CD11b^+^ cells were derived from aGVHD mice with the treatment of IL-22Ab or IgG. Cells were cocultured in 48-well plates with IL-2 (50 IU/ml, R&D) and anti-CD3 (20 ng/ml, R&D) for 3 days. RPMI 1640 medium was supplemented with 100 units/ml penicillin, 100 mg/ml streptomycin, 10 mM HEPES (Invitrogen Life Technologies), and 10% heat-inactivated fetal calf serum (Hyclone) and was used for all cultures. Foxp3 expression was determined by flow cytometry. In some groups, the cells were stimulated with IL-22 (100 ng/ml) or DMSO. The proliferation of T cells was tested through flow cytometry.

### 2.7. Western Blot Analysis

Proteins were extracted from harvested cells, and their concentration was determined by the BCA assay (Pierce). Protein samples (30 *μ*g) were resolved by SDS-PAGE and transferred to a PVDF membrane. The following antibodies were used: IL-22R1 (ABCAM, ab211675), Stat1 (Cell Signaling Technology, number 9172), Stat3 (Cell Signaling Technology, number 9139), p-Stat1 (Cell Signaling Technology, number 9167), and p-Stat3 (Cell Signaling Technology, number 9145). The results were visualized with a Kodak autoradiography film (Kodak XAR film).

### 2.8. Suppressive Assay In Vitro

CD4^+^CD25^−^ T cells were selected from normal F1 mice, labeled with carboxyfluorescein succinimidyl ester (CFSE), and cultured with CD11b^+^ cells obtained from aGVHD mice on day 7 with the treatment of IL-22Ab or IgG at a ratio of 5 : 1. In some groups, the cells were stimulated with IL-22 (100 ng/ml, R&D) or DMSO on day 0. The proliferation of T cells was tested by flow cytometry. All flow cytokines were purchased from BioLegend. For the cytokine test, cells were stimulated with PMA/ionomycin for 4 hours and brefeldin A for 1 hour before intracellular cytokine staining. Briefly, cells were fixed for 1 hour and permed (eBioscience) for 10 min before cytokine staining.

### 2.9. Statistical Analysis

Data are represented as the means ± SEM. Statistical comparisons with the indicated group were performed by the Mann–Whitney *U* test or ANOVA using GraphPad Prism software. Differences in Kaplan-Meier survival curves were analyzed by the log-rank test. *p* < 0.05 was considered to be significant.

## 3. Results

### 3.1. IL-22 Expression Was Upregulated in the Mouse aGVHD Model

**Figure 1:**
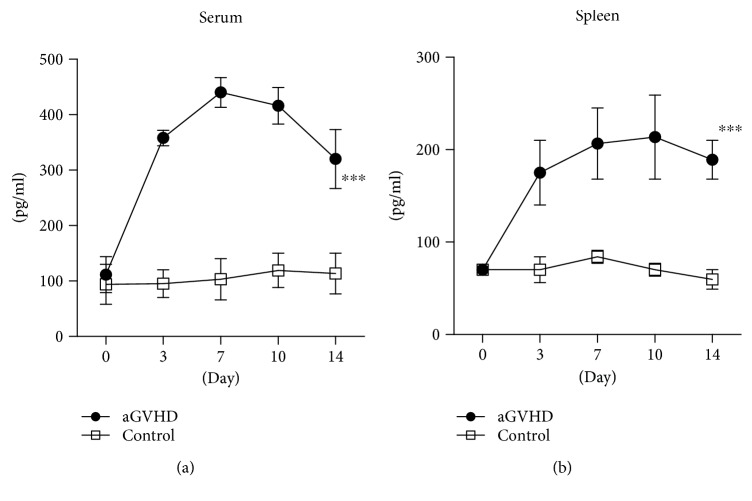
IL-22 expression was increased in aGVHD mice. aGVHD was induced as described in Materials and Methods. Mice were sacrificed, and the serum and spleen were collected. IL-22 expression in (a) serum and (b) spleen cells was tested by ELISA. Data are shown as the mean ± SEM from five independent experiments. ^∗∗∗^ *p* < 0.001.

To investigate the role of IL-22 in the pathogenesis of aGVHD, we compared the pattern of IL-22 expression in normal and aGVHD mice. D2B6F1 mice were injected intravenously with 50 × 10^6^ B6 spleen cells (Figure S1A), and serum IL-22 levels were measured at various time points using ELISA and compared with levels in nontransplant mice. In mice injected with B6 spleen cells, the concentration of IL-22 increased, reaching a peak on day 10 posttransplantation, then slowly declining thereafter (Figure 1(a)).

We also examined IL-22 expression in spleen cells after transplantation of B6 cells by ELISA using homogenates prepared from the spleen of normal and aGVHD mice. The pattern of IL-22 expression in the spleen was similar to that in the serum (Figure 1(b)). These data thus clearly show that IL-22 is involved in the pathogenesis of aGVHD.

### 3.2. Infusion of IL-22Ab Markedly Suppressed Donor Cell Engraftment and Prevented the Disease-Associated Depletion of Host Cells in aGVHD Mice

**Figure 2:**
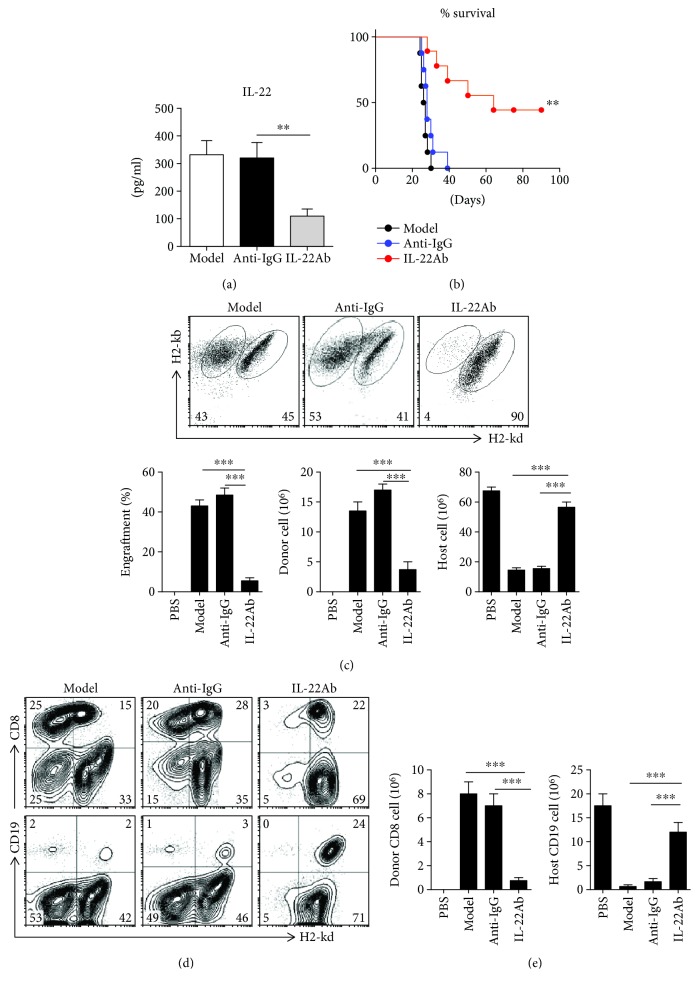
Infusion of IL-22Ab markedly suppressed engraftment of donor cells in aGVHD mice. aGVHD was induced as described in Materials and Methods. Mice were treated with PBS, anti-IgG, or IL-22Ab. After 2 weeks, mice were sacrificed, and donor and host splenic lymphocyte subsets were analyzed by flow cytometry. Splenocytes were stained with H2-Kd and H2-Kb to distinguish donor and host cells. (a) IL-22 expression in serum on day 14 for each group was tested by ELISA. (b) Survival of aGVHD mice in each group. (c) Representative plots and relative engraftment percentages and absolute numbers of donor and host cells after 14 days. (d) Representative plots of donor and host CD8^+^ and CD19^+^ cells. (e) Absolute numbers of donor CD8^+^ cells and host CD19^+^ cells shown as total numbers. Data are shown as the mean ± SEM from six independent experiments. ^∗∗^ *p* < 0.01 and ^∗∗∗^ *p* < 0.001.

To determine whether treatment with IL-22Ab prevents the development of aGVHD, B6 spleen cells (50 × 10^6^) were injected intravenously into immunocompetent D2B6F1 mice. In some groups, IL-22Ab (20 *μ*g/mouse, ProSpec, Israel) was coinjected with B6 spleen cells. Control mice received coinjection of B6 cells and anti-IgG (20 *μ*g/mouse, ProSpec) (Figure S1A). Mice were sacrificed on day 14, and serum and spleen cells were collected and examined. Serum levels of IL-22 were suppressed on day 14 in mice injected with IL-22Ab (Figure 2(a)). The survival of mice injected with the IL-22Ab was significantly prolonged compared with the aGVHD and anti-IgG groups, whereas there was no difference between the anti-IgG and the aGVHD groups (Figure 2(b)). Donor and host cells were distinguished by staining with H2-Kd and H2-Kb, as donor cells were H2-Kb^+^/d^−^ and host cells were H2-Kb^+^/d^+^. At 2 weeks after cell transfer, nearly 50% of the cells in aGVHD mice were donor cells (Figure 2(c)), which was consistent with a previously reported parent- (B6-) to-F1 aGVHD model [16]. Interestingly, injection of IL-22Ab inhibited the engraftment of donor cells. A reduction in the number of lymphocytes in the host is a major characteristic of aGVHD [22]. Although the transfer of B6 spleen cells led to a reduction in the number of total spleen cells in F1 mice and host cells, cotransfer of IL-22Ab significantly inhibited donor cell expansion and the reduction in the number of host cells, whereas cotransfer of control anti-IgG did not (Figure 2(c)).

Next, we examined the phenotypes and frequencies of donor and host cells in the aGVHD and IL-22Ab groups. In the B6-to-F1 model, donor CD8^+^ cells play an important role in initiating aGVHD [23]. Infusion of IL-22Ab markedly suppressed the expansion of donor CD8^+^ cells (Figures 2(d) and 2(e)). The killing of host B cells by activated donor CD8^+^ cells is another characteristic of B6-to-F1 aGVHD [23]. As shown in Figures 2(d) and 2(e), the percentage and total number of CD19^+^ B cells declined dramatically in aGVHD mice compared with normal F1 mice. Conversely, the cotransfer of IL-22Ab almost completely inhibited the reduction in the number of host B cells compared with cotransfer of anti-IgG. Thus, our data demonstrate that IL-22Ab suppresses the expansion of donor CD8^+^ cells and inhibits the cytotoxic effects of donor CD8^+^ cells on host CD19^+^ cells.

### 3.3. Infusion of IL-22Ab Suppressed Apoptosis, Increased the Expression of Foxp3, and Reduced Cytokine Expression *In Vivo*

**Figure 3:**
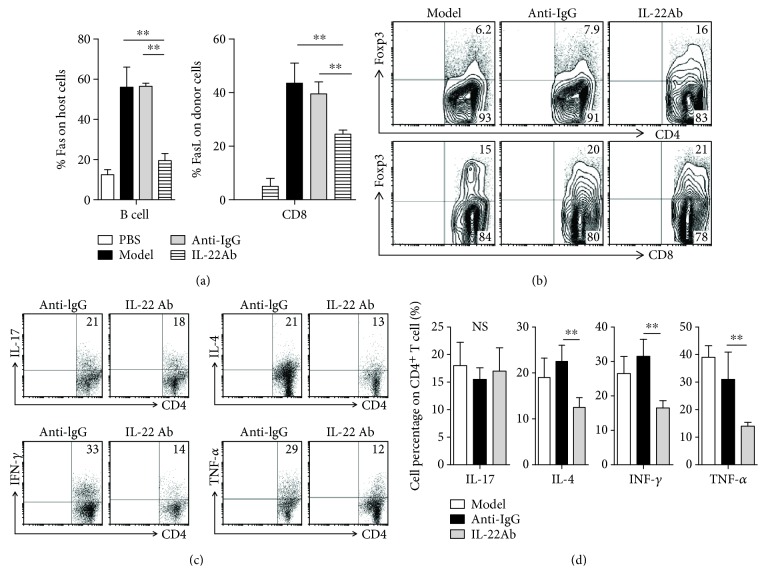
Infusion of IL-22Ab suppressed cell apoptosis, increased the expression of Foxp3, and reduced cytokine expression *in vivo*. (a) At 14 days after cell transfer, Fas expression in host B cells and FasL expression in donor CD8^+^ T cells from the spleen were examined by flow cytometry. (b) aGVHD was induced as previously described. After 14 days, mice were sacrificed, and the spleen cells were stained for forkhead box p3 (Foxp3), CD4, and CD8. Representative contour plots of CD4^+^Foxp3^+^ and CD8^+^Foxp3^+^ cells determined by flow cytometry. Data are shown as the means from five independent experiments. Each group included five mice. (c, d) Representative plots of percentages of CD4^+^ T cells expressing various cytokines, including IL-17, IL-4, interferon- (IFN-) *γ*, and tumor necrosis factor- (TNF-) *α*, as determined by flow cytometry. Bars show the mean ± SEM of six independent experiments. ^∗∗^ *p* < 0.01.

The Fas/FasL interaction is thought to be important in the induction of apoptosis in aGVHD via the death-inducing signaling complex [24]. We examined the levels of Fas and FasL expression in both host and donor cells in aGVHD mice using flow cytometry. Levels of Fas expression in host B cells were significantly elevated in mice in the model and anti-IgG groups compared with PBS-injected mice. Cotransfer of IL-22Ab markedly inhibited the upregulation of Fas expression in host B cells. FasL expression on CD8^+^ donor cells increased following aGVHD induction; however, injection of IL-22Ab significantly suppressed the upregulation of FasL expression on donor CD8^+^ cells (Figure 3(a)).

To determine the role of Foxp3 expression in the protective effect of IL-22Ab treatment in aGVHD mice, we examined levels of Foxp3 following treatment with anti-IgG or IL-22Ab. Cotransfer of IL-22Ab increased the percentage of Foxp3-expressing CD4^+^ cells and slightly increased the percentage of CD8^+^Foxp3^+^ cells (Figure 3(b)). It is likely that IL-22Ab treatment also increases the number of CD4^+^Foxp3^+^ cells, thereby indirectly protecting against the development of aGVHD.

Previous studies have suggested that increased cytokine production contributes to the pathogenesis of aGVHD. To determine whether IL-22Ab suppresses the development of aGVHD by modulating cytokine production, we evaluated CD4^+^ T cells expressing various cytokines potentially associated with the observed decreased severity of aGVHD on day 14. Injection of aGVHD mice with IL-22Ab led to significantly reduced percentages of CD4^+^ cells secreting proinflammatory cytokines such as interferon- (IFN-) *γ*, IL-4, and tumor necrosis factor- (TNF-) *α*. No difference in the number of CD4^+^ cells expressing IL-17 was detected, however (Figures 3(c) and 3(d)). CD4^+^ cells expressing other Th2 cytokines, such as IL-5 and IL-13, were undetectable in this model, and IL-22Ab treatment did not alter their percentages (data not shown). To confirm the flow cytometry data, we also sorted CD4^+^ T cells from the spleen of mice from different groups on day 14. The cells were cultured in 1640 medium for 12 hours, and then cytokine levels in the supernatant were assayed. The analyses showed that CD4^+^ T cells derived from IL-22Ab-treated mice expressed significantly lower levels of IFN-*γ*, IL-4, and TNF-*α* compared with cells derived from the anti-IgG-treated mice, in agreement with the flow cytometry data (Figure S2).

### 3.4. Tregs Were Shown to Mediate IL-22-Induced aGVHD Protection

**Figure 4:**
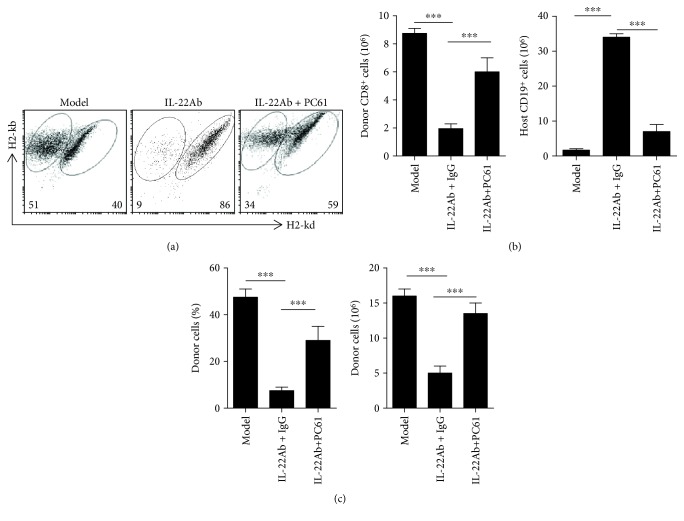
Foxp3^+^ regulatory T cells play an important role in protecting against the deleterious effects of IL-22 in aGVHD. D2B6F1 mice were injected intraperitoneally with PC61 (anti-CD25 monoclonal antibody; 250 g/mouse) or control IgG simultaneous with transfer of B6 cells. aGVHD was induced as previously described. After 14 days, mice were sacrificed, and donor cells and host cells were enumerated using flow cytometry. (a) Representative plots of donor and host cells 14 days after cell transfer. (b) Number of donor CD8^+^ and host B cells. (c) Relative engraftment percentage and total number of donor cells. Data are shown as the mean ± SEM from five independent experiments. ^∗∗∗^ *p* < 0.001.

The importance of Tregs in IL-22Ab-mediated inhibition of aGVHD development was examined by administering a monoclonal antibody against CD25 (CD25MoAb or PC61) to abolish the function of Tregs [25, 26]. Mice were injected intraperitoneally with PC61 (250 g/mouse) or control IgG on the same day they were injected with 50 × 10^6^ B6 cells. After 14 days, the mice were sacrificed, and the spleen cells were examined by flow cytometry. The effect of IL-22Ab on aGVHD was generally reversed after treatment with PC61 (Figure 4(a)), consistent with the survival data shown in Figure S1A. Both IL-22Ab-mediated suppressions of donor CD8^+^ cell function and expansion and protection of host B cells and total host cells were significantly abolished upon PC61 treatment (Figure 4(b)).

### 3.5. IL-22Ab Treatment Increased Foxp3 Expression through CD11b^+^ Cells

**Figure 5:**
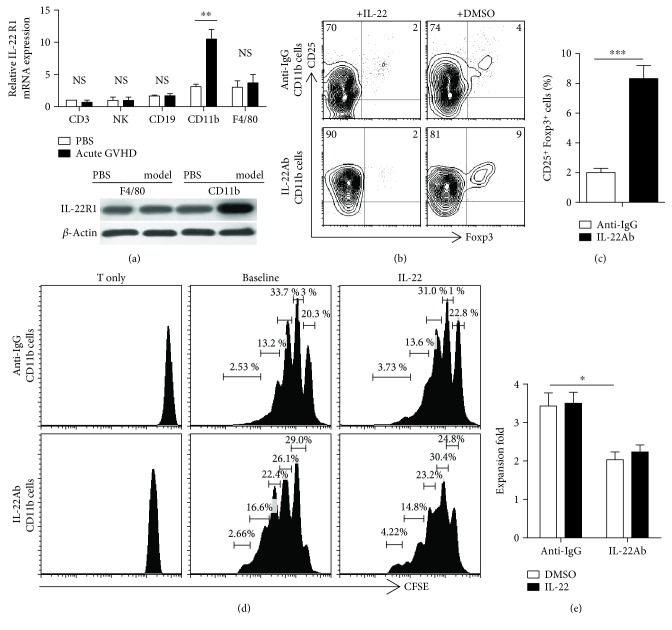
IL-22Ab treatment increased Foxp3 expression and suppressed T cell proliferation via CD11b^+^ cells. CD11b^+^ cells were obtained from the spleen of anti-IgG- or IL-22Ab-treated aGVHD mice by positive selection using anti-PE-CD11b and anti-PE beads (Miltenyi Biotec). CD4^+^CD25^−^ T cells were obtained from the spleen of normal B6 mice. The two cell subsets were cocultured at a ratio of 1 : 5 for 3 days. In some experiments, IL-22 was replenished to verify its function in T cell differentiation and expansion. (a) Relative expression of IL-22R1 mRNA in CD3^+^ T cells, natural killer (NK) cells, CD19^+^ cells, CD11b^+^ cells, and F4/80^+^ cells in normal D2B6F1 mice and aGVHD mice (day 7). The CD3 cell vehicle of the PBS group was arbitrarily assigned a value of 1, with other values presented relative to this value. The expression of IL-22R1 on CD11b^+^ and F4/80^+^ cells derived from aGVHD mice on day 7 was determined by Western blotting. (b) Representative contour plots of CD25^+^Foxp3^+^ cells with the addition of TGF-*β* and IL-2; cells were gated on CD4^+^ T cells 3 days after culture. DMSO: dimethyl sulfoxide. (c) Relative number of CD25^+^Foxp3^+^ T cells among CD4^+^ cells. (d) Representative histogram plots of T cell expansion after coculture with aGVHD model-derived CD11b^+^ cells in the presence of anti-CD3 with and without IL-22 for 3 days. (e) Proliferation capacity of each cell group. Data are shown as the mean ± SEM from four independent experiments. ^∗^ *p* < 0.05, ^∗∗^ *p* < 0.01, and ^∗∗∗^ *p* < 0.001.

Next, we determined which immune cell subsets respond to IL-22. IL-22R1 is the major receptor mediating the function of IL-22 [27]. The interaction between IL-22R1 and IL-22 is thought to contribute to synovial inflammation in rheumatoid arthritis [28]. Intestinal epithelial cells, CD3^+^ T cells, natural killer cells, CD19^+^ cells, CD11b^+^ cells, and F4/80^+^ macrophages were sorted from the spleen of normal and aGVHD mice, and the expression of IL-22R1 mRNA was evaluated using RT-PCR. Expression of IL-22R1 mRNA was slightly higher in CD11b^+^ and F4/80^+^ cells compared with the other cell subsets. aGVHD induction enhanced IL-22R1 mRNA expression without affecting the number of F4/80^+^ cells *in vivo*. We therefore examined the expression of IL-22R1 via Western blotting, which showed that IL-22R1 was elevated in CD11b^+^ cells (but not F4/80^+^ cells) only upon induction of aGVHD (Figure 5(a)).

CD11b^+^ cells are important antigen-presenting cells. As we demonstrated that treatment with IL-22Ab increases Foxp3 expression in CD4^+^ T cells, we then examined whether IL-22Ab contributes to the expansion of CD4^+^Foxp3^+^ cells via CD11b^+^ cells *in vitro*. CD11b^+^ cells were selected from aGVHD mice treated with and without IL-22Ab or anti-IgG 1 week after aGVHD was established. Compared with the anti-IgG group, IL-22Ab-treated CD11b^+^ cells exhibited stronger induction of CD4^+^Foxp3^+^ cell expansion. When IL-22 was reintroduced, the percentage of T cells expressing Foxp3 declined (Figures 5(b) and 5(c)). We also examined the ability of CD11b^+^ cells to induce the activation and expansion of T cells *in vitro*. CD11b^+^ cells and CD4^+^CD25^−^ T cells were derived as described above. CD4^+^CD25^−^ T cells were labeled with CFSE, and the two cell subsets were cocultured at a ratio of 1 : 5 in the presence of anti-CD3 antibody. Additionally, PC61 was added to neutralize the activity of Tregs. In comparison with the anti-IgG group, T cell proliferation was strongly suppressed when CD11b^+^ cells from the IL-22Ab-treated group were cocultured with T cells (Figures 5(d) and 5(e)). We added IL-22 to examine whether the effect of anti-IgG would be reversed; however, no differences were observed when IL-22 was added during cell culture in either group.

### 3.6. IL-22Ab Inhibited the Pathogenesis of aGVHD via Donor CD11b^+^ Cells

**Figure 6:**
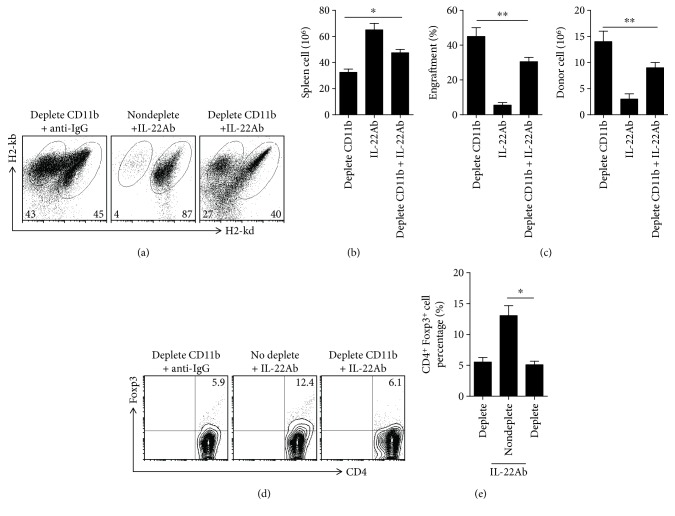
IL-22Ab inhibited the pathology of aGVHD via donor CD11b^+^ cells. CD11b^+^ cells were depleted among B6 spleen cells, and the remaining spleen cells were injected into D2B6F1 mice. After 14 days, mice were sacrificed, and the donor and host splenic lymphocyte subsets, as well as the expression of Foxp3 in CD4^+^ T cells, were analyzed using flow cytometry. (a) Representative plots of donor and host cells after 14 days. (b) Absolute number of total cells. (c) Relative engraftment percentage and absolute number of donor cells. (d) Representative contour plots of CD4^+^Foxp3^+^ T cells. (e) Relative number of CD25^+^Foxp3^+^ T cells among CD4^+^ cells. Data are shown as the mean ± SEM from six independent experiments. ^∗^ *p* < 0.05, ^∗∗^ *p* < 0.01, and ^∗∗∗^ *p* < 0.001.

To investigate the role of CD11b^+^ cells in the protective effect of IL-22Ab against aGVHD, CD11b-depleted B6 spleen cells were injected into F1 mice. CD11b depletion abolished the protective effect of IL-22Ab in aGVHD mice (Figure S1B). Mice were sacrificed on day 14, and the spleen cells were examined by flow cytometry. The effect of IL-22Ab on aGVHD was generally reversed in CD11b-depleted mice (Figure 6(a)). In addition, CD11b depletion significantly abolished the protective effect on host cell survival, decrease in donor cell engraftment, and suppression of donor cell expansion associated with IL-22Ab treatment (Figures 6(b) and 6(c)). We also examined the percentage of Foxp3-expressing CD4^+^ cells in each donor subset group. Although cotransfer of IL-22Ab increased the percentage of CD4^+^ cells expressing Foxp3, Foxp3 was not elevated in the CD11b-depleted group with or without IL-22Ab (Figures 6(d) and 6(e)).

### 3.7. IL-22Ab Regulated the Phenotype and Function of CD11b^+^ Cells in aGVHD Mice

**Figure 7:**
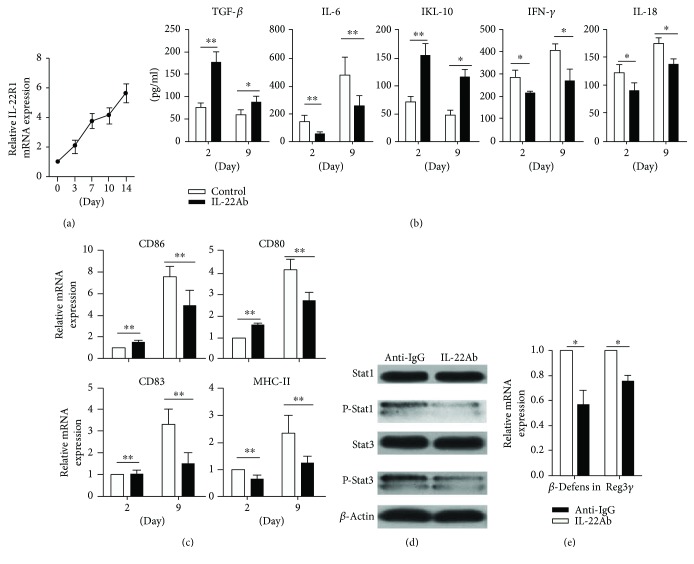
IL-22Ab regulated the phenotype and function of CD11b^+^ cells in aGVHD mice. (a) Relative expression of IL-22R1 mRNA in CD11b^+^ cells from aGVHD mice at different time points. The day 0 vehicle was arbitrarily assigned a value of 1, with the other values shown relative to this value. (b) Expression of TGF-*β*, IL-6, IL-18, IFN-*γ*, and IL-18 in CD11b^+^ cells from aGVHD mice treated with anti-IgG or IL-22Ab on days 2 and 9. (c) Relative expression of CD80, CD86, CD83, and MHC-II mRNA in CD11b^+^ cells from aGVHD mice treated with anti-IgG or IL-22Ab on days 2 and 9 examined using RT-PCR. The day 2 vehicle in the anti-IgG group was arbitrarily assigned a value of 1, with the other values shown relative to this value. (d) The expression of Stat1, P-Stat1, Stat3, and P-Stat3 in CD11b^+^ cells examined using Western blotting. (e) Levels of *β*-defensin and Reg3*γ* mRNA in CD11b^+^ cells examined using RT-PCR. The anti-IgG vehicle was arbitrarily assigned a value of 1, with the other values shown relative to this value. Data are shown as the mean ± SEM from four independent experiments. ^∗^ *p* < 0.05 and ^∗∗^ *p* < 0.01.

Finally, we analyzed the expression of receptors at different time points in CD11b^+^ cells derived from mice in the aGVHD group on days 0, 3, 7, 10, and 14. The expression of IL-22R1 mRNA was analyzed by RT-PCR. Interestingly, IL-22R1 expression increased over time, peaking on day 9 (Figure 7(a)). CD11b^+^ cells from aGVHD mice treated with anti-IgG or IL-22Ab were isolated on days 2 and 9, and the production of cytokines and expression of costimulatory molecules were evaluated by ELISA and RT-PCR. CD11b^+^ cells produced significantly lower amounts of proinflammatory cytokines such as IL-6, IFN-*γ*, and IL-18 but higher amounts of IL-10 and TGF-*β* on day 2 and day 9 in the IL-22Ab-treated group (Figure 7(b)). Moreover, increased levels of CD86, CD80, CD83, and MHC-II were observed on day 9 compared with day 2 (Figure 7(c)).

Inflammatory cytokines exert their effects via the phosphorylation of Stat1 and Stat3 [29–31]. We hypothesized that IL-22Ab treatment would lead to reduced levels of Stat1 and Stat3 phosphorylation in mice with aGVHD initiated by IL-22. Although CD11b^+^ cells from aGVHD model mice treated with and without IL-22Ab showed similar levels of Stat1 and Stat3 expression, as expected, IL-22Ab treatment led to markedly decreased phosphorylation of Stat1 (p-Stat1) and Stat3 (p-Stat3) in CD11b^+^ cells derived from aGVHD mice (Figure 7(d)). Furthermore, as previous reports demonstrated that upregulation of *β*-defensin and Reg3*γ* is required for IL-22-mediated immune regulation [32, 33], we evaluated the expression of *β*-defensin and Reg3γ by CD11b^+^ cells obtained from aGVHD mice with and without IL-22Ab treatment using RT-PCR. The data showed that IL-22Ab treatment led to reduced expression of both *β*-defensin and Reg3*γ* in CD11b^+^ cells compared with cells from mice treated with anti-IgG. Thus, we conclude that IL-22Ab inhibits inflammation at least in part via the regulation of *β*-defensin and Reg3*γ* expression.

## 4. Discussion

Previous studies have found that IL-22 plays differing roles in the pathogenesis of a number of autoimmune diseases. For example, *Helicobacter*-induced increased IL-22 expression protects mice from acute pancreatitis [32], and IL-22 mediates the protective effect of *γδ* T cells in lung fibrosis [34]. By contrast, administration of IL-22Ab protects against systemic lupus erythematosus [35] and rheumatoid arthritis [1]. Here, we show that the impact of IL-22 on hematopoietic cells differs compared with the effect on tissues.

This study addressed the role of IL-22Ab in regulating CD8^+^ cell expansion and CTL effect of donor cells in a p-F1 aGVHD mouse model. Our results show that IL-22 is highly expressed in the serum and spleen cells after aGVHD induction. IL-22Ab was administered to neutralize the effect of IL-22. As expected, intravenous injection of D2B6F1 mice with IL-22Ab significantly reduced the severity of aGVHD. The killing effect of CD8^+^ cells plays an important role in the pathogenesis of aGVHD [23]; however, we found that IL-22Ab-treated mice harbored fewer donor CD4^+^ and CD8^+^ cells compared with untreated aGVHD mice. Furthermore, a higher percentage of host CD19^+^ cells was observed in mice treated with IL-22Ab, indicating that IL-22Ab suppresses the killing of host B cells by donor T cells. IL-22Ab-treated mice also exhibited fewer IFN-*γ* ^+^ and TNF-*α* ^+^ T cells, both of which play significant inflammatory roles in the progression of aGVHD [36, 37]. IL-22 expression can result in the phosphorylation of JAK2/Stat3 [38], initiating the production of IFN-*γ* or TNF-*α* [39, 40]. In the present study, IL-22Ab treatment was shown to increase the number of CD4^+^Foxp3^+^ cells in aGVHD mice, in agreement with data reported by another group [17]. When mice were injected with PC61 (anti-CD25) to deplete the Treg population, the protective role of IL-22Ab was diminished, confirming that IL-22Ab protects against aGVHD by enhancing Foxp3^+^ expression by T cells.

IL-22 exerts its biological functions via IL-22R1 and IL-10R2. On the cell surface, these receptors are adjacent and overlap [41, 42]. Because IL-10R2 is widely expressed by a variety of cell types and binds to both IL-10 and IL-22, the selective effect of IL-22 is determined primarily by the expression of IL-22R1 on the surface of specific cells, such as epithelial cells [43], keratinocytes [44], and hepatocytes [45]. CD11b^+^ cells (but no other type of immune cell) express IL-22R1 after allostimulation [46]. To study the role of CD11b^+^ cells in the protective function of IL-22Ab against aGVHD, CD11b-depleted donor spleen cells were isolated and injected into host mice. As expected, IL-22Ab protected the host CD11b^+^ cells only in the presence of donor CD11b^+^ cells. We also analyzed the number of CD4^+^Foxp3^+^ T cells *in vivo*. CD4^+^ Tregs were reduced in number in the CD11b-depleted group, even after treatment with IL-22Ab. These data show that IL-22Ab protects against aGVHD by modulating CD11b^+^ cells to increase the number of CD4^+^ Tregs. Furthermore, higher levels of Foxp3 expression were observed in CD4^+^CD25^+^ T cells cocultured with CD11b^+^ cells in the presence of IL-22Ab. CD11b^+^ antigen-presenting cells from IL-22Ab-treated mice affected the expansion of pathogenic T cells to a much lesser degree than normal antigen-presenting cells.

Importantly, our data demonstrate that administration of IL-22Ab protects against aGVHD by increasing the number of Foxp3^+^ cells. Although Couturier et al. also found that deficient IL-22 production by donor T cells attenuates murine aGVHD [17], Hanash et al. reported that IL-22 deficiency results in increased aGVHD-associated tissue damage [19]. To explain this contradiction, we evaluated the expression of IL-22 receptors on the surface of CD11b^+^ cells at different time points after aGVHD induction. IL-22R1 expression peaked on day 9 but decreased during the later phases of aGVHD. To evaluate the relationship between CD11b and IL-22Ab treatment, CD11b^+^ cells were derived from aGVHD mice that were treated with anti-IgG or IL-22Ab on days 2 and 9. On day 9, IL-22Ab-treated CD11b^+^ cells secreted lower amounts of IL-6, a proinflammatory cytokine involved in the differentiation of naïve T cells into antigen-specific effector Th1 and Th17 cells, both of which play important roles in the pathogenesis of aGVHD [47]. CD11b^+^ cells from IL-22Ab-treated mice also expressed higher amounts of IL-10 and TGF-*β*, which mediate the expansion and function of Tregs [48, 49]. Higher expression of these two cytokines might be associated with decreased expression of positive costimulatory molecules.

Hanash et al.'s group reported that recipient-derived IL-22 deficiency is associated with greater aGVHD-associated tissue damage and mortality, a finding that differs fundamentally from our findings [19]. This discrepancy could be explained as follows: first, the aGVHD model used by the two groups was different. Hanash et al. transferred B6 donor marrow and T cells into lethally irradiated BALB/c recipient mice, almost 75% of which died less than 10 days after the model was established. In our model, by contrast, spleen cells of B6 mice were transferred to B6D2F1 mice that were not subjected to lethal irradiation. Mortality began in these mice 20 days posttransplantation, indicating that the tissue damage in the B6-BALB/c model mice was more severe compared with that of the B6-DBA2F1 model mice prior to modulation of CD11b^+^ cells (IL-22Ab effect on CD11b^+^ cells on day 9 is shown in Figure 7) and expansion of Tregs. Second, although the mechanism underlying the effects observed in each model differed, there was no contradiction between the models in that expression of IL-22 receptors on different cells which led to different effects. For example, Hanash et al.'s group reported that IL-22 functions as a critical regulator of tissue sensitivity to GVHD via activation of IL-22R1 expressed on intestinal stem cells, whereas our group determined that treatment with IL-22Ab increased the number of Foxp3^+^ T cells regulating IL-22R1 expressed on CD11b^+^ cells.

**Figure 8:**
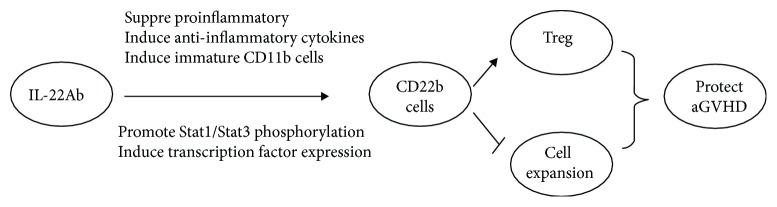
IL-22Ab regulates the phenotype and function of CD11b^+^ cells to induce Treg expansion and suppress donor-specific CD8^+^ T cell expansion and CTL activity to protect against aGVHD.

Because we used nonirradiated donor D2B6F1 mice, we were able to observe the expansion of donor cells and the killing effect of donor CD4^+^ and CD8^+^ cells on host CD19^+^ cells. However, as marked tissue damage, weight loss, and death are rare during the early phase of aGVHD, we focused on the effect of IL-22 on CD11b^+^ antigen-presenting cells. Three mechanisms may explain the protective effect of IL-22Ab via CD11b^+^ cells: (1) IL-22Ab treatment modulated the expression of inflammatory cytokines involved in the pathogenesis of aGVHD, reducing the expression of IL-6, IFN-*γ*, and IL-18 and enhanced that of IL-10 and TGF-*β*. These results suggest that IL-22Ab protects against aGVHD in part via modulation of cytokine expression. (2) Compared with mice treated with anti-IgG, expression of costimulatory molecules such as CD80, CD86, and MHC-II was downregulated in mice treated with IL-22Ab. We also examined the expression of CD83, an important marker of mature dendritic cells (DCs). CD83, which is expressed on the DC membrane, enhances immune responses by boosting intracellular calcium release in T lymphocytes [50]. We hypothesized that IL-22Ab regulates the transformation of mature DCs to immature DCs. (3) The levels of P-Stat1 and P-Stat3 and IL-22-related transcription factors such as *β*-defensin and Reg3*γ* were downregulated following administration of IL-22Ab. Thus, expansion of Foxp3^+^ T cells was induced, and the proliferation of effector T cells was inhibited both *in vitro* and *in vivo* (Figure 8).

In summary, our results demonstrated that IL-22Ab modulates the function of CD11b^+^ cells, resulting in the differentiation of Tregs and suppression of donor-specific CD8^+^ T cell expansion and CTL activity to protect against aGVHD (Figure 8). As CD11b is expressed by a wide variety of myeloid cells, a further study should focus on identifying the cellular target of IL-22 and determining which CD11b^+^ cell subsets play the most important role in IL-22Ab-associated aGVHD protection. Although IL-22 exerts both pro- and anti-inflammatory effects, our findings suggest that IL-22 is an inflammatory cytokine in GVHD. Our research indicates that inhibition or blockade of IL-22 and its axis of interacting molecules are both a feasible and beneficial strategy for treating GVHD.

## Data Availability

The data used to support the findings of this study are available from the corresponding author upon request.

## Abbreviations

aGVHD: Acute graft-versus-host disease
Foxp3: Forkhead box P3
Tregs: Regulatory T cells.

## Supplementary Materials

Supplementary Materials Figure S1: (A) schematic of IL-22Ab injection in the in vivo aGVHD model. (B) Survival of aGVHD mice treated with IL-22 with and without PC61. (C) Survival of aGVHD mice treated with IL-22Ab with and without depletion of CD11b+ cells. Kaplan-Meier survival curves depict the percentage of live mice (*n* = 8 for each group). ^∗∗∗^ *p* < 0.001. Figure S2: cytokine expression in CD4+ T cell supernatants as determined using ELISA. CD4+ T cells were sorted using bead selection on day 14 for each group and cultured for 12 hours before the supernatants were collected. Data are shown as the mean ± SEM from three independent experiments. NS indicates no significant difference; ^∗^ *p* < 0.05.
