# Supplementary material for: Anti-IL-22 Antibody Attenuates Acute Graft-versus-Host Disease via Increasing Foxp3+ T Cell through Modulation of CD11b+ Cell Function
Source: J Immunol Res. 2018 Aug 7;2018:1605341. doi: 10.1155/2018/1605341 (PMC6109487; doi:10.1155/2018/1605341)
Supplement: Supplementary Materials — Figure S1: (A) schematic of IL-22Ab injection in the in vivo aGVHD model. (B) Survival of aGVHD mice treated with IL-22 with and without PC61. (C) Survival of aGVHD mice treated with IL-22Ab with and without depletion of CD11b+ cells. Kaplan-Meier survival curves depict the percentage of live mice (n = 8 for each group). ∗∗∗ p < 0.001. Figure S2: cytokine expression in CD4+ T cell supernatants as determined using ELISA. CD4+ T cells were sorted using bead selection on day 14 for each group and cultured for 12 hours before the supernatants were collected. Data are shown as the mean ± SEM from three independent experiments. NS indicates no significant difference; ∗ p < 0.05. [file 1605341.f1.docx]

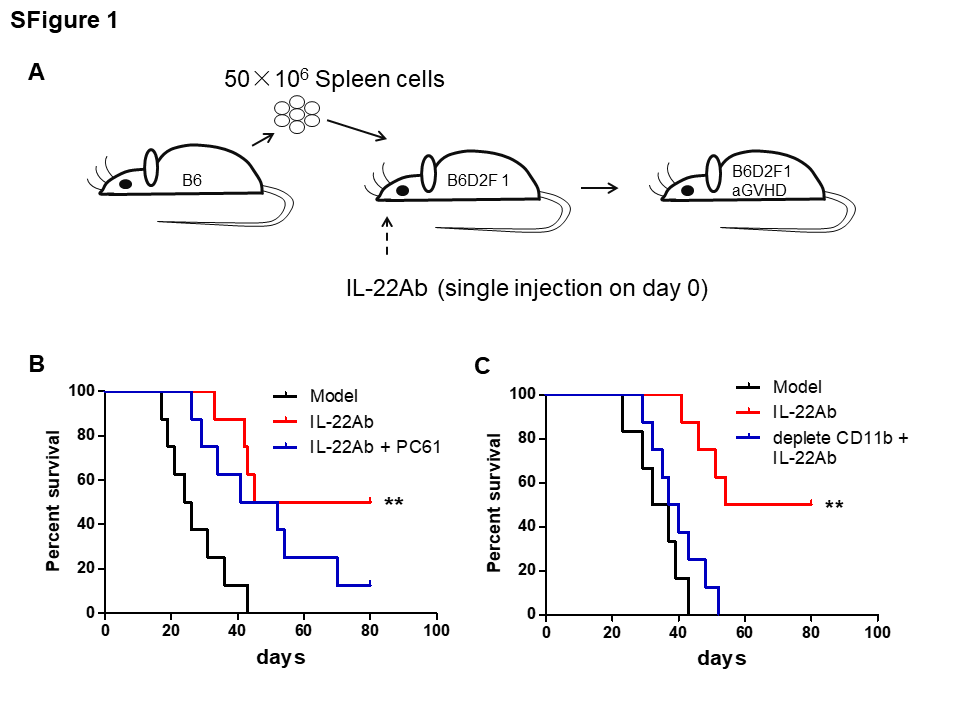


**SFigure 1.** (A) Schematic of IL-22Ab injection in the *in vivo* aGVHD model. (B) Survival of aGVHD mice treated with IL-22 with and without PC61. (C) Survival of aGVHD mice treated with IL-22Ab with and without depletion of CD11b^+^ cells. Kaplan-Meier survival curves depict the percentage of live mice (n=8 for each group). **p<0.01.


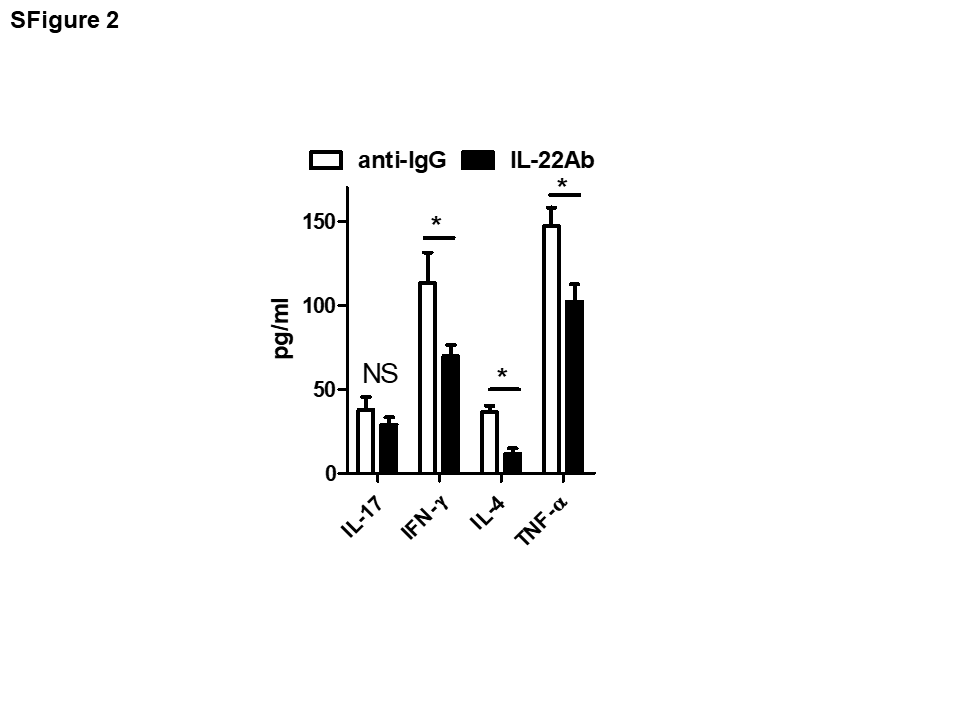


**SFigure 2.** Cytokine expression in CD4^+^ T-cell supernatants as determined using ELISA. CD4^+^ T cells were sorted using bead selection on day 14 for each group and cultured for 12 hours before the supernatants were collected. Data are shown as the mean ± SEM from three independent experiments. NS indicates no significant difference; * p<0.05.
